# Genomic Variations Explorer (GenVarX): a toolset for annotating promoter and CNV regions using genotypic and phenotypic differences

**DOI:** 10.3389/fgene.2023.1251382

**Published:** 2023-10-09

**Authors:** Yen On Chan, Jana Biová, Anser Mahmood, Nicholas Dietz, Kristin Bilyeu, Mária Škrabišová, Trupti Joshi

**Affiliations:** ^1^ MU Institute for Data Science and Informatics, University of Missouri-Columbia, Columbia, MO, United States; ^2^ Department of Biochemistry, Faculty of Science, Palacky University in Olomouc, Olomouc, Czechia; ^3^ Division of Plant Science and Technology, University of Missouri-Columbia, Columbia, MO, United States; ^4^ Plant Genetics Research Unit, United States Department of Agriculture-Agricultural Research Service, Columbia, MO, United States; ^5^ Christopher S. Bond Life Sciences Center, University of Missouri-Columbia, Columbia, MO, United States; ^6^ Department of Electrical Engineering and Computer Science, University of Missouri-Columbia, Columbia, MO, United States; ^7^ Department of Biomedical Informatics, Biostatistics and Medical Epidemiology, University of Missouri-Columbia, Columbia, MO, United States

**Keywords:** transcription factor, promoter, copy number variation, whole genome re-sequencing data, genomic variations, SNPs, Indels, phenotypes

## Abstract

The rapid growth of sequencing technology and its increasing popularity in biology-related research over the years has made whole genome re-sequencing (WGRS) data become widely available. A large amount of WGRS data can unlock the knowledge gap between genomics and phenomics through gaining an understanding of the genomic variations that can lead to phenotype changes. These genomic variations are usually comprised of allele and structural changes in DNA, and these changes can affect the regulatory mechanisms causing changes in gene expression and altering the phenotypes of organisms. In this research work, we created the GenVarX toolset, that is backed by transcription factor binding sequence data in promoter regions, the copy number variations data, SNPs and Indels data, and phenotypes data which can potentially provide insights about phenotypic differences and solve compelling questions in plant research. Analytics-wise, we have developed strategies to better utilize the WGRS data and mine the data using efficient data processing scripts, libraries, tools, and frameworks to create the interactive and visualization-enhanced GenVarX toolset that encompasses both promoter regions and copy number variation analysis components. The main capabilities of the GenVarX toolset are to provide easy-to-use interfaces for users to perform queries, visualize data, and interact with the data. Based on different input windows on the user interface, users can provide inputs corresponding to each field and submit the information as a query. The data returned on the results page is usually displayed in a tabular fashion. In addition, interactive figures are also included in the toolset to facilitate the visualization of statistical results or tool outputs. Currently, the GenVarX toolset supports soybean, rice, and *Arabidopsis*. The researchers can access the soybean GenVarX toolset from SoyKB via https://soykb.org/SoybeanGenVarX/, rice GenVarX toolset, and *Arabidopsis* GenVarX toolset from KBCommons web portal with links https://kbcommons.org/system/tools/GenVarX/Osativa and https://kbcommons.org/system/tools/GenVarX/Athaliana, respectively.

## Introduction

As the use of sequencing technology becomes popular in both industrial and academic sectors, a large amount of whole genome re-sequencing (WGRS) data has become publicly available for users to utilize for research activities or commercial purposes. From the WGRS data, researchers can understand the genomic variations and the potential effects on phenotypes of organisms (Bolger et al., 2017). The differences in phenotypes compared among accessions are the reflections of genomic variations and structural variations (Li et al., 2022). The genomic variations include changes of alleles in gene regions, upstream promoter regions, and intergenic regions, while the structural changes comprise insertion, deletion, and duplication of DNA segments. These allele changes can occur in the transcription factor binding domain which leads to regulatory mechanism dysfunction for some target genes. Ultimately, the issues cause alterations in gene expression patterns and lead to phenotypic diversity in organisms. Similarly, the larger structural variations seen in the form of copy number variations (CNVs) can cause gains or losses in DNA segments. These modifications in DNA can alter the copies of expressed genes and ultimately results in changes in phenotypes in organisms (Żmieńko et al., 2014). Thus, gaining an in-depth understanding of the transcription factor (TF) binding sites and the CNVs through performing analysis with extensive data and open-source tools and packages that are available online is critical in plant research and development.

Currently, there are more than 3000 soybean, 1000 *Arabidopsis*, and 3000 rice WGRS accession datasets along with phenotype datasets, annotation datasets, and transcription factors datasets scattered across different resources (The 3000 rice genomes project, 2014; Alonso-Blanco et al., 2016; Liu et al., 2020). The datasets are usually available for researchers to download in the form of static files and are not pre-integrated with other omics datasets. The publicly accessible web portals and platforms such as Plant Transcription Factor Database (PlantTFDB) (Jin et al., 2016), Plant Transcriptional Regulatory Map (PlantRegMap) (Tian et al., 2019), Gene Transcription Regulation Database (GTRD) (Yevshin et al., 2017), and JASPAR (Castro-Mondragon et al., 2021) are the main providers of transcription factors related datasets. Moreover, the National Center for Biotechnology Information (NCBI), European Nucleotide Archive (ENA), the Genome Sequence Archive (GSA) of the National Genomics Data Center (NGDC), and the CyVerse data store (Goff et al., 2011; Merchant et al., 2016) are the main resources for publicly available WGRS datasets. Likewise, there are also many open-source tools and packages available that can be applied to the WGRS data and used to perform CNV analysis like cn.MOPS (Klambauer et al., 2012), CNV-seq (Xie and Tammi, 2009), cnvScan (Samarakoon et al., 2016), and more. Nevertheless, there is a lack of interactive and visualizable web applications to integrate and query TF binding sites in promoter regions and CNVs data with the genomic variability observed from large-scale studies involving thousands of accessions to gain insight about phenotypes, perform validations, and eventually roll out new discoveries. To solve this problem, we have dedicated effort towards building an interactive and visualization-enhanced toolset for supporting this analysis.

## Materials and methods

In the materials and methods section, we provide details about the datasets collected and utilized for this research work including the tools used to obtain the datasets, process the data, and the respective required inputs and outputs for these tools. The processed datasets are uploaded and stored in the MySQL database integrated into both SoyKB (Joshi et al., 2012; Joshi et al., 2013; Joshi et al., 2017) and KBCommons (Zeng et al., 2018; Zeng et al., 2019) web portals. The package used for uploading the datasets, indexing methods for the tables in the database, and technology used in the web development for the GenVarX toolset are also described.

### Datasets

The GenVarX toolset currently supports the soybean, rice, and *Arabidopsis* organisms. For each organism, the toolset is divided into two components mainly for the promoter regions and the CNV analysis. In order to facilitate the functionalities of each component in each organism, many datasets are required to be collected and processed together to power the GenVarX toolset.

For the promoter regions component, the transcription factors (TFs) datasets and binding TF position probability matrices from the Plant Transcription Factor Database (PlantTFDB v5.0) (Jin et al., 2016) were acquired for each organism. In addition, the predicted transcription factor binding sites datasets and motif-gene regulation datasets from the Plant Transcriptional Regulatory Map (PlantRegMap) website (Tian et al., 2019) were required for each organism.

For the CNV analysis component, we acquired WGRS datasets for each organism (in different formats depending on their availability in public resources) and generated the CNV results. The WGRS files mainly include sequencing data in FASTQ format and the reads mapped to genome sequences in Binary Alignment Map (BAM) format. The FASTQ format datasets are further processed and converted into BAM files as the BAM files are the required input format for our selected tool for generating CNV results.

For the CNV analysis component in soybean, we have acquired WGRS datasets of 1066 distinct soybean accessions from publicly available datasets including Zhou302v2 (Zhou et al., 2015), Liu304 (Liu et al., 2020), USB-15x (Valliyodan et al., 2021), USB-40x (Valliyodan et al., 2021), Soja (Kim et al., 2010), and MSMC (Valliyodan and Nguyen, 2006). The PGen pipeline (Liu et al., 2016) was used for mapping these reads to Williams 82 version 2 (Wm82.a2.v1) reference genome and for SNP and Indel calling to generate both BAM and Variant Call Format (VCF) files. The mapped soybean sequencing reads in BAM format for all datasets were collectively stored on the Cyverse Data Store. The soybean Wm82.a2.v1 reference genome was downloaded from the Phytozome website (Goodstein et al., 2011). We have also collected soybean phenotype datasets from the Germplasm Resources Information Network (GRIN) database.

Similarly, we have acquired 3000 BAM files and VCF files of rice from the 3000 Rice Genome on Amazon Web Services (AWS) (2014). The Nipponbare reference genome that was used to generate these BAM files was downloaded from the Rice Annotation Project Database (RAP-DB) (Sakai et al., 2013). Regarding the rice phenotypic datasets, our research group acquired the datasets from the International Rice Research Institute’s official web portal (https://snp-seek.irri.org/_download.zul).

Furthermore, a selected set of 1043 *Arabidopsis* FASTQ files have also been acquired from the SRA Run Selector with project number PRJNA273563 using the SRA Toolkit 3.0.0 (https://github.com/ncbi/sra-tools). These *Arabidopsis* accessions are part of the 1135 *Arabidopsis* accessions presented in the 1001 Genomes Consortium (Alonso-Blanco et al., 2016). Since the collected files are in FASTQ format, we have also collected the *Arabidopsis thaliana* TAIR10 reference genome from the Phytozome website for mapping the FASTQ files to the reference genome and generating the BAM files. We also directly downloaded the *Arabidopsis* VCF files from the 1001 Genomes Consortium (https://1001genomes.org/data/GMI-MPI/releases/v3.1/).

### Data processing

In data processing, the datasets for the promoter regions component and the CNV analysis component of the GenVarX toolset were processed separately using a different set of open-source and publicly available tools. There are four types of datasets that are utilized in the promoter regions component. The datasets are TF datasets, predicted transcription factor binding sites datasets, motif-gene regulation datasets, and binding TF position probability matrices. The first three types of datasets only need some additional processing for extracting the mandatory columns that are used in the promoter regions component. The binding TF position probability matrices, on the other hand, are processed further using the Ceqlogo tool in the Meme Suite (Bailey et al., 2015). The details about the binding TF position probability matrices and the sequence logo figures will be utilized in the promoter regions component to overlap with genomic variations (SNP and Indels) datasets and show the potential impacts of nucleotide variations on conserved positions in motifs.

For the CNV analysis component, there were two types of datasets involved, the FASTQ and BAM files. The FASTQ files were required to be processed and converted into BAM files so that they can be used as inputs to the cn. MOPS package. In the data processing of the FASTQ files, the files were aligned with their respective reference genome using the Burrows-Wheeler Aligner tool (BWA) version 0.7.17 (Li and Durbin, 2009) to create outputs in Sequence Alignment Map (SAM) format. Moreover, the SortSam, MarkDuplicates, and AddOrReplaceReadGroups commands in the Genome Analysis Toolkit (GATK) version 4.2.6.1 (McKenna et al., 2010) were used to sort the SAM files, mark duplications, assign reads to read groups, and output the final BAM files required for the next step.

In order to generate the CNV results, we utilized the cn. MOPS R package (Klambauer et al., 2012) written in C++ and R by Klambauer et al. (2012). The cn. MOPS package can take in BAM files to calculate the coverage depth of each position across accessions and perform read variations decomposition based on the mixture components and Poisson distributions across accessions using a Bayesian method (Klambauer et al., 2012). Because of its implementation, this package can achieve a low false discovery rate (FDR) as high noise data is filtered out during the calculation process. The cn. MOPS package has demonstrated good performance and significance using metrics such as the precision-recall area-under-curve (PR AUC) and recall rate by comparing itself with other methods. The outputs of this package that were useful to our CNV analysis component are the CNV individual hits of each accession and the CNV consensus regions across all samples.

For the analysis, we have written R scripts to perform CNV calculations for each organism separately. Each R script that uses the cn. MOPS package takes in the file paths of the BAM files as inputs. The R script was designed to perform CNV analysis on the chromosomal sequences of an organism. Other non-chromosomal sequences like scaffold sequences, mitochondria sequences, and more were not included in the CNV analysis. Upon the completion of CNV analysis, CNV individual hits and consensus regions were collected and ready to be uploaded to the database.

### Data storing

In the data storing section, we provide details about database, data upload methods, and data indexing methods. Uploading data into databases and storing the data with proper indexing can enhance the data query by speeding up the process and preserving the data for long-term usage. In our GenVarX toolset development, we adopted this common practice in order to provide good services to users.

In the GenVarX toolset development, the MySQL database integrated into the SoyKB and KBCommons web portals was utilized to store the datasets for both the promoter regions component and the CNV analysis component of different organisms. With regard to uploading the datasets to the MySQL database, the open-source SQLAlchemy package (Bayer, 2012) written in Python was used to assist the data upload process. For datasets that are very large in size such as the CNV consensus regions datasets and genotype datasets, the B+ tree indexing method was used to index the tables in the database. Having all necessary datasets in the database, queries from the web applications can be facilitated as the data in the database can be searched and returned to the users.

### Web development

The GenVarX toolset is presented as a web-based application to the users. Therefore, the user-interactive parts of the GenVarX toolset were web-focused. In web development, several programming languages, libraries, and frameworks were utilized. Because the soybean GenVarX toolset and the GenVarX toolset of other organisms are being deployed on different platforms, the used frameworks are slightly different between the two GenVarX toolsets.

In the development of the soybean GenVarX toolset, HTML, CSS, JavaScript, PHP, and SQL programming languages were utilized in coding both the promoter and the CNV analysis components. Among the four programming languages, HTML, CSS, and JavaScript were focused on the front-end of the web application, while PHP and SQL were focused on the back-end development. In the front-end of the web application, the jQuery JavaScript library was used for enhancing the JavaScript functions and sending requests to the back-end for data retrieval. The PHP back-end of the web application was focused on rendering PHP code and communicating with the database to collect data from the database using SQL queries. The entire technological structure is closer to Linux, Apache, MySQL, and PHP (LAMP) stack.

Likewise, the GenVarX toolsets for other organisms also used the same programming languages, and the programming languages were focused on the front-end and back-end, respectively, similar to the soybean GenVarX toolset. The jQuery JavaScript library was also used for the same purposes in the GenVarX toolsets for the other organisms. Nevertheless, the framework used in the GenVarX toolsets for the other organisms was the Laravel framework which encompasses the back-end of the web application in classes that extends the based controller class and manages all the routes of the web application in one place. Using the Laravel framework, the GenVarX toolset can work for many organisms in the same code. Hence, using this framework simplifies the web development processes.

The deployment of the soybean GenVarX toolset and the other universal GenVarX toolsets are on different websites. The soybean GenVarX toolset was deployed on the SoyKB website while the rest of the toolsets were deployed on the KBCommons website. The links to access the GenVarX toolset are placed under the tool section of both websites. Users can click on the links to get redirected to the GenVarX toolset. For simplicity, below are the links to access the different GenVarX toolsets hosted on the SoyKB and KBCommons websites:

• Soybean GenVarX toolset: https://soykb.org/SoybeanGenVarX/
• Rice GenVarX toolset: https://kbcommons.org/system/tools/GenVarX/Osativa
• *Arabidopsis* GenVarX toolset: https://kbcommons.org/system/tools/GenVarX/Athaliana

## Results

In the results section, the promoter regions and the CNV analysis components of the GenVarX toolset are illustrated. The results focus on the functionalities, interfaces, inputs, and outputs of each component of the GenVarX toolset.

### Promoter regions component

The promoter regions component consists of a data search page, an independent promoter results page, and a phenotype data viewing page. The promoter search windows on the data search page allow users to search by gene IDs or search by binding TFs. Both windows take in user inputs to perform queries and render the results on the promoter results page and the phenotype data viewing page. Here, each search method is discussed separately.

### Promoter regions component—Search by Gene IDs

**FIGURE 1:**
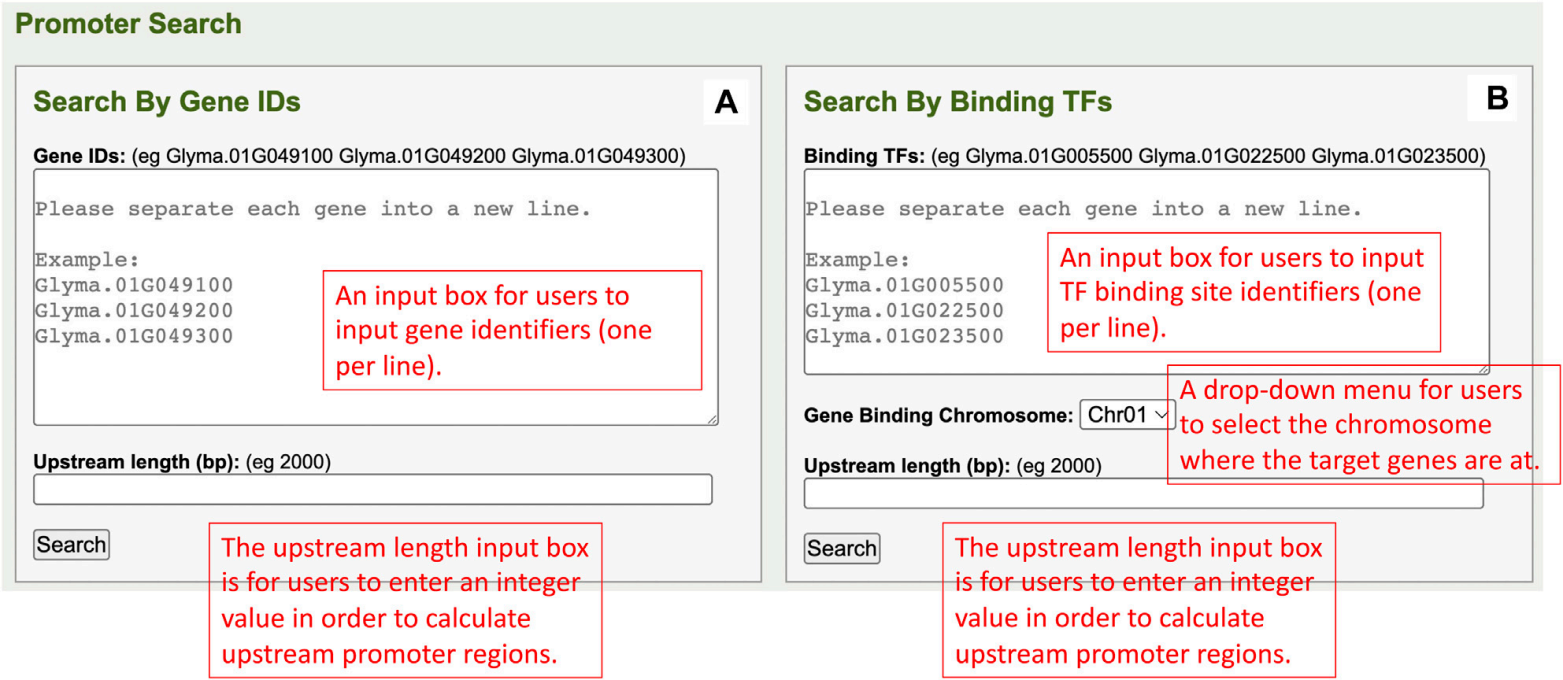
**(A)** The Search by Gene IDs window in the promoter regions component. **(B)** The Search by Binding TFs window in the promoter regions component.

In the Search by Gene IDs of the promoter regions component, there is a window that has one gene identifiers input box, an upstream length input box, and a search button (Figure 1A). The input box allows users to input multiple gene identifiers at one time, and each gene identifier must be separated into a new line. The upstream length input box takes an integer value from users to calculate upstream promoter regions of the inputted genes. When the search button is clicked, the query is done for each gene, that is, searchable in the database. At the same time, TF binding sites that are in the promoter region and all relevant information are also fetched from the database and displayed on the promoter results page.

**FIGURE 2:**
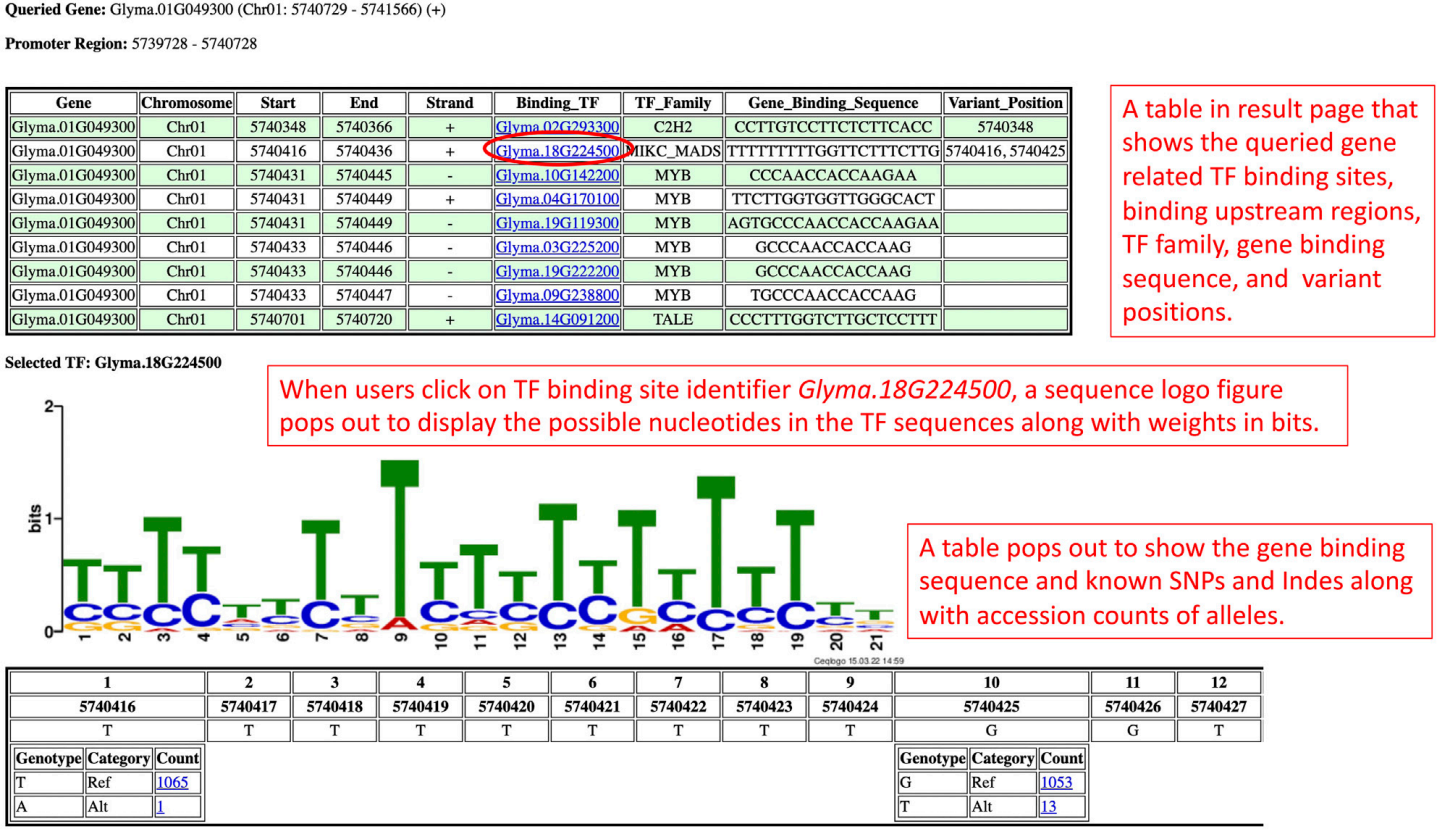
The promoter results page is rendered based on the query from the Search by Gene IDs section. This results page presents the TF binding sites of a gene in a table along with the sequence logo figure and position-nucleotide table.

On the promoter results page, the TF binding sites results are grouped by genes and shown independently for one section per gene (Figure 2). Each section begins with the queried gene identifier along with the chromosome number, coordinates, and strand information. Below the gene identifier, a calculated upstream promoter region is shown. Underneath that, a TF binding sites table with information by row for each TF binding site in the promoter region displays information such as the TF binding site chromosome number, coordinates, strand, TF binding site identifiers, TF family type, and Williams 82 version 2 gene binding sequence. Each TF binding site identifier in the table is a clickable hyperlink. Users can click on the TF binding site identifier that they are interested in to retrieve more details about that TF binding site.

When users click on a TF binding site identifier (Binding_TF), a sequence logo figure and a position-nucleotide table will be loaded onto the respective section of the promoter results page. From the sequence logo figure, users can visualize the possible nucleotides in that TF binding site along with the information entropy of each nucleotide calculated using the Shannon entropy formula (Schneider and Stephens, 1990). The table below the sequence logo figure shows each position and each nucleotide of the Williams 82 version 2 gene binding sequence in a tabular form to assist in comparing the nucleotides in the Williams 82 version 2 gene binding sequence with the possible nucleotides of the TF binding sites shown in the sequence logo figures. The comparison provides insight into the sequence conservation of the nucleotides in the Williams 82 version 2 gene binding sequence and the TF binding sites.

**FIGURE 3:**
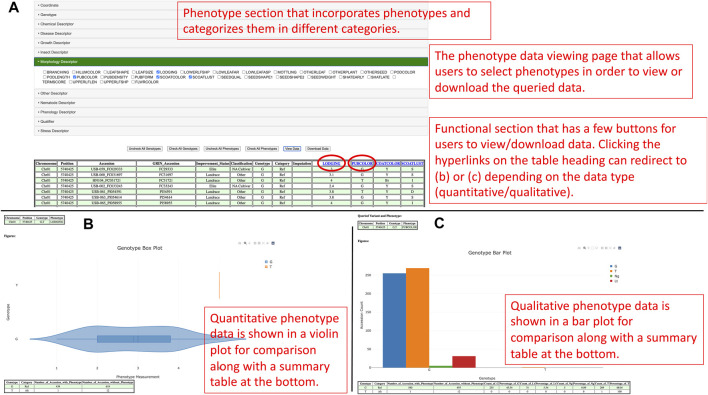
**(A)** The phenotype data viewing page for users to select genotypes and phenotypes they are interested in and view or download the data. **(B)** The distribution of quantitative phenotype data plotted against genotypes in a violin plot figure. **(C)** The distribution of qualitative phenotype data plotted against genotypes in a bar plot.

Apart from that, the GenVarX toolset also shows SNPs and Indels in allele tables along with the counts of accessions having the corresponding alleles. The counts on the table are clickable and able to redirect users to the phenotype data viewing page (Figure 3A). On the phenotype data viewing page, users not only can see accessions that have a particular allele but also able to connect the accessions with phenotype data. In the phenotype accordion drop-down menu, users can select the phenotypes in order to view or download the phenotype data. The phenotype headings on the table are also clickable to plot violin plots or bar plots depending on the data type (quantitative or qualitative) of that phenotype column (Figures 3B, C).

The purpose of generating violin plots and bar plots is to show the distributions of the phenotype data for different alleles. From a violin plot, users can understand the quantitative distribution of phenotype data based on the maximum, minimum, first quantile, third quantile, mean, and median values of that box. Likewise, users can uncover the qualitative distribution of phenotype data in a bar plot according to the counts of different qualitative categories. From the plots, users can also potentially understand the significance and linkage between alleles and phenotypes. Besides the plots, summary tables are also provided at the bottom of each figure to summarize the counts of accessions based on genotypes and either the existence of phenotype data for quantitative measurement or the categories of qualitative measurement. At the bottom of the page, users can also visualize the distribution of improvement status of accessions in different genotypes to understand the linkage between phenotype, improvement status, and genotypes.

### Promoter regions component—Search by Binding TFs

The Search by Binding TFs of the promoter regions component has a window, that is, composed of a TF binding site input box, a gene binding chromosome dropdown list, an upstream length input box, and a search button (Figure 1B). In the TF binding site identifier input box, users can input multiple TF binding site identifiers with each in a new line. The gene-binding chromosome drop-down menu allows users to select one chromosome per search. The upstream length input box is for users to input an integer value for upstream promoter regions of genes’ calculations. When users click on the search button, the user input information is utilized in performing queries, and the results are returned to the promoter results page.

**FIGURE 4:**
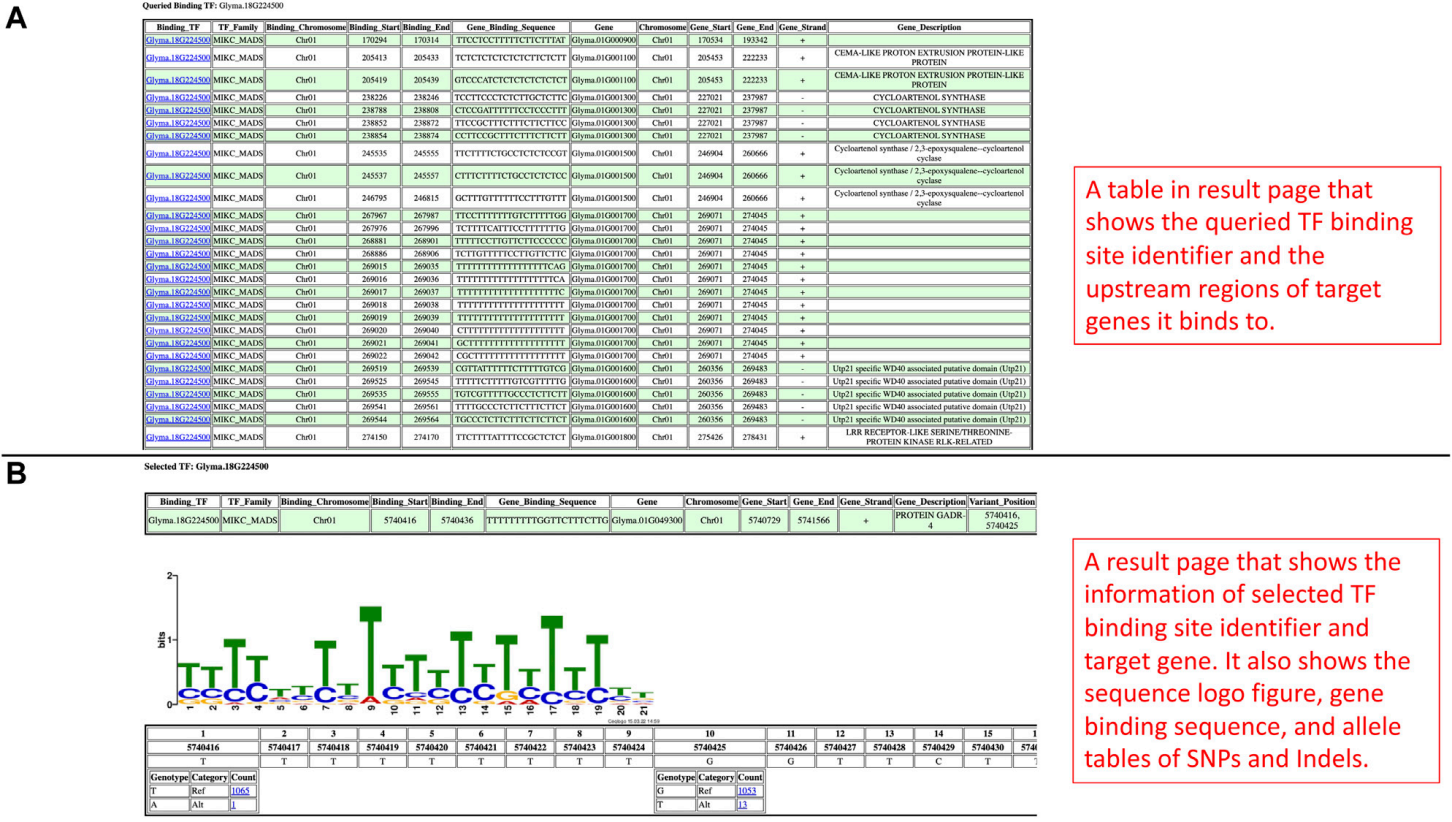
**(A)** The promoter results page is rendered based on the query from the Search by Binding TFs section. It includes tables showing the information on TF binding sites and target genes. **(B)** This page is shown when users select a TF binding site identifier. This page has information related to the selected TF binding site, sequence logo figure, and position-nucleotide table.

In the promoter results page, the information of each TF binding site identifier and its corresponding regulating genes are shown as an independent table (Figure 4A). In each table, the TF binding site identifier is clickable to redirect to a new page to display a sequence logo figure, position-nucleotide table, as well as SNPs and Indels in allele tables (Figure 4B). Similar to the results page in the Search by Gene IDs section, the alleles can also be linked with phenotype data in the phenotype data viewing page, and the functionalities such as data viewing, data downloading, and data plotting are also included as well (Figures 3A–C).

### Copy number variation (CNV) component

The CNV analysis component consists of three different search sections which are the Search by Gene IDs section, Search By Accession and Copy Numbers section, and Search by Chromosome and Region section. Each section has a search window for users to input data for queries and the results of the queries are rendered on the corresponding results pages. Here, each section is discussed in more detail.

### CNV analysis component—Search by Gene IDs

**FIGURE 5:**
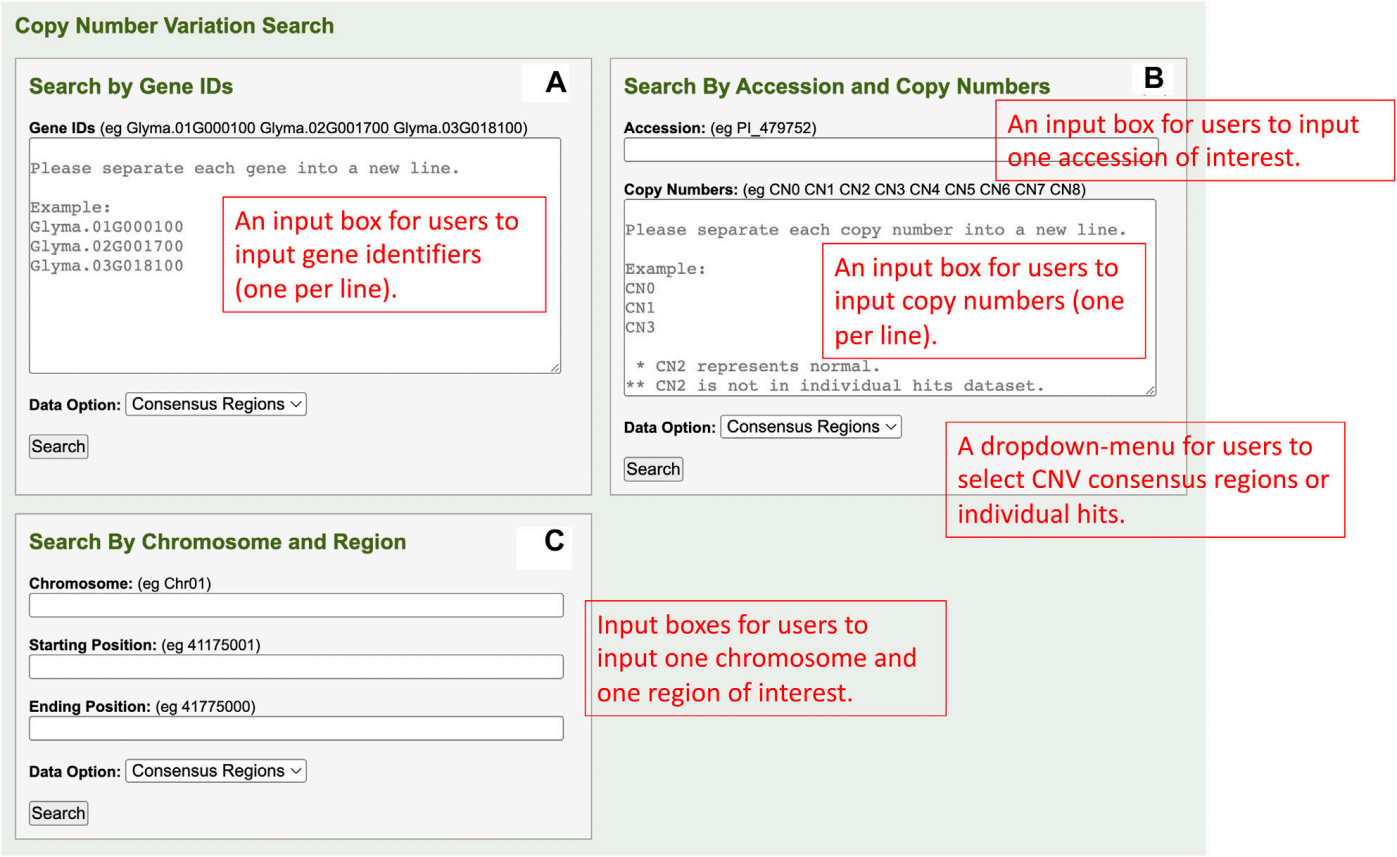
**(A)** The Search by Gene IDs window in the CNV analysis component. **(B)** The Search By Accession and Copy Numbers window in the CNV analysis component. **(C)** The Search by Chromosome and Region window in the CNV analysis component.

In the Search by Gene IDs of the CNV analysis component, there is one gene ID input box, a data option dropdown menu, and a search button in a window (Figure 5A). Users can input multiple genes of interest into the gene IDs input box, and each gene ID has to be separated into a new line. In the data option dropdown menu, users can select either consensus regions or individual hits. The consensus regions option is for CNV data summarized across accessions, and the individual hits option is for the CNV individual region of each accession. After the user completes the inputs, the user can click on the search button to perform queries and redirect to the results page.

**FIGURE 6:**
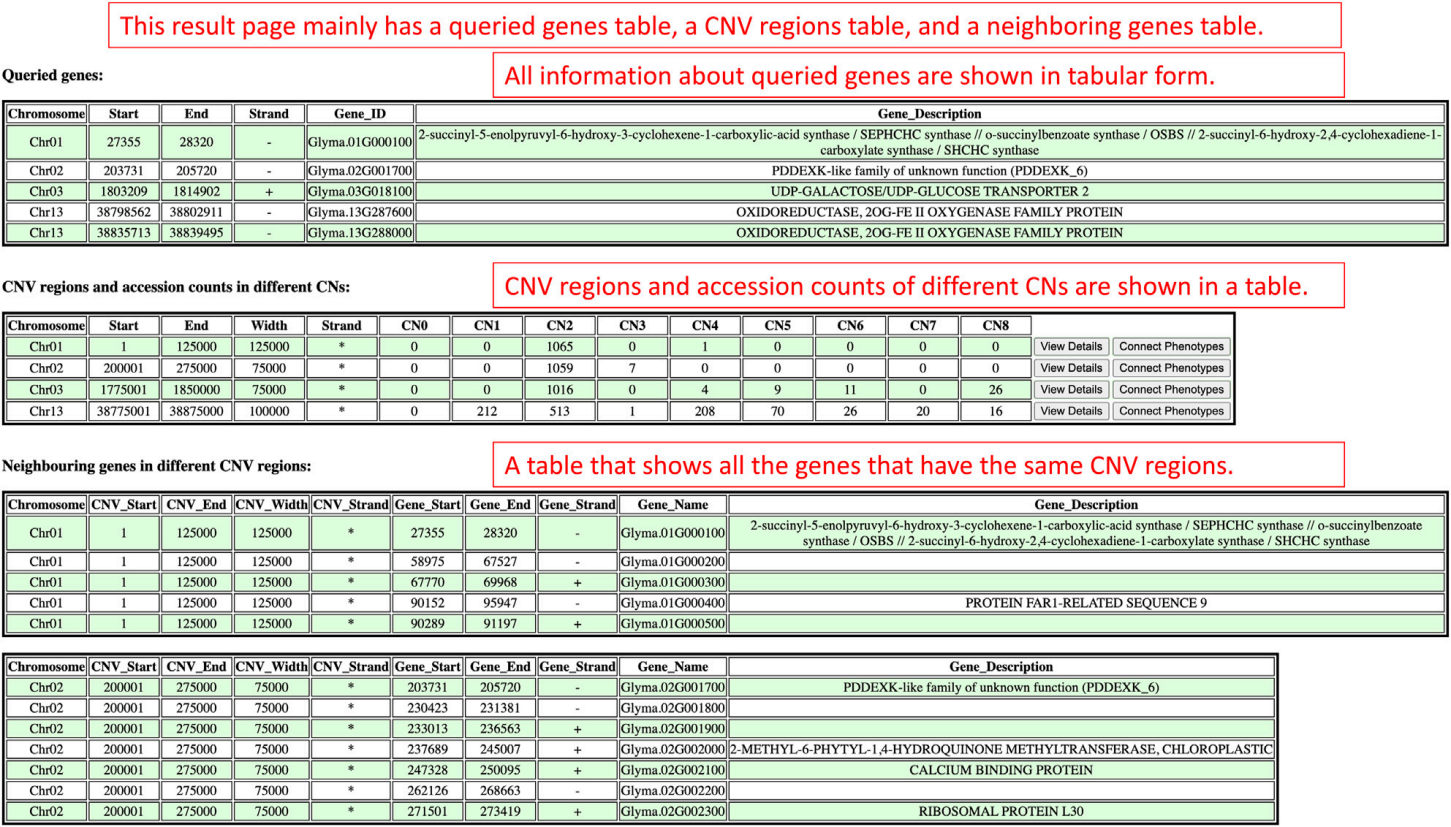
The results render on the results page when users use the Search by Gene IDs window in the CNV analysis component.

There are three sections on the Search by Gene IDs’ results page which are the queried genes section, the CNV regions and accession counts section, and the neighboring genes in different CNV regions section (Figure 6). The queried genes section displays genes’ relevant information like chromosomes, coordinates, strands, identifiers, and descriptions. Based on the coordinates of the queried genes, CNV regions that enclosed the queried gene coordinates are displayed in the CNV regions and accession counts section as a table along with the counts of accessions within copy numbers (CN0–CN8). According to the cn. MOPS tool, CN0 and CN1 represent loss, CN2 is normal, and CN3 to CN8 represent gain (Klambauer et al., 2012). Each CN region and the accession counts within copy numbers are organized in a row. At the end of each row, there are view details button and connect phenotypes button that can be clicked to redirect to the detail viewing page and phenotype data viewing page. In the neighboring genes in different CNV regions section, each CNV region and the genes within that CNV region are shown as an independent table.

**FIGURE 7:**
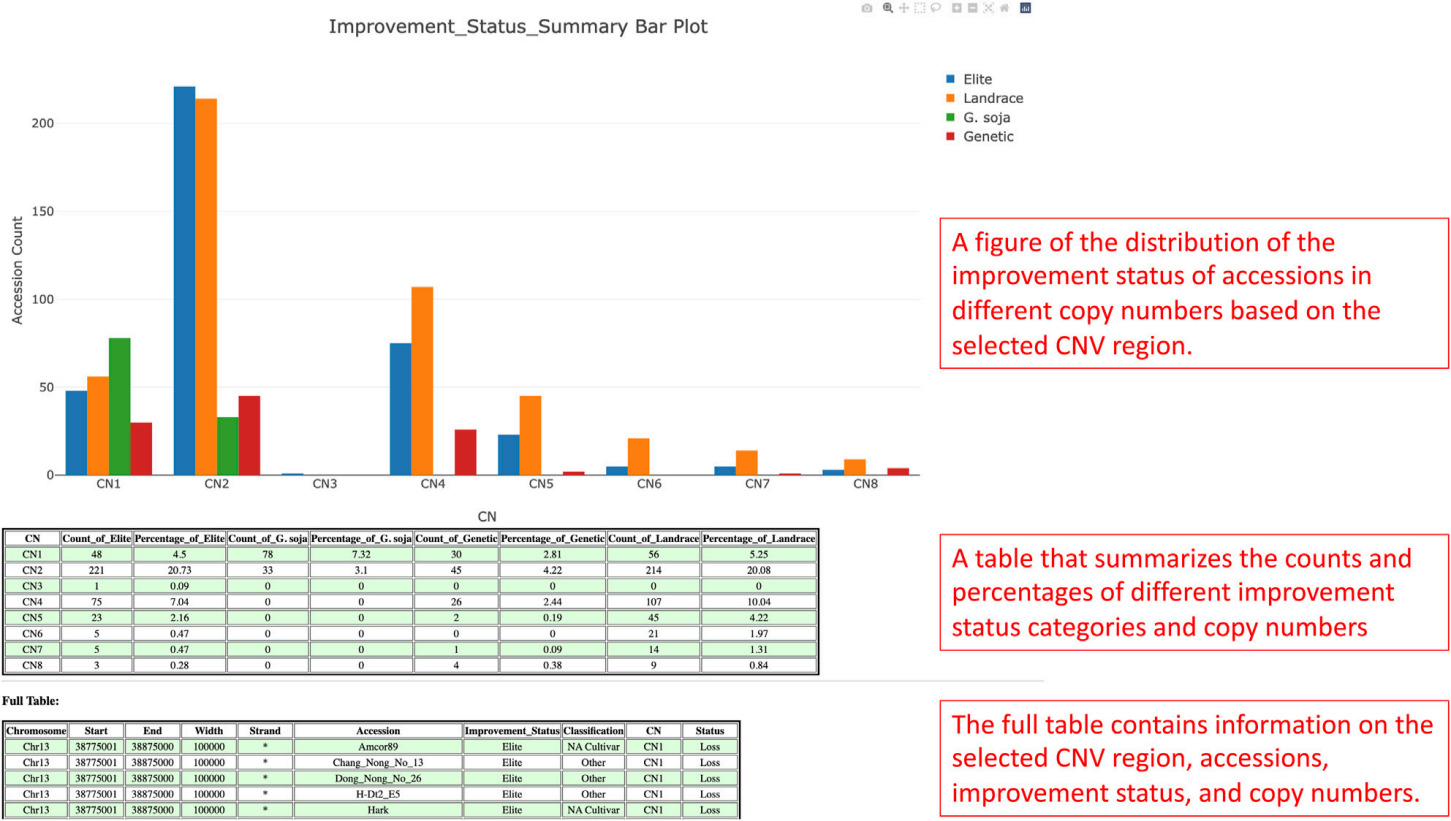
The detail viewing page shows the distribution of the improvement status of the selected CNV region along with a summary table of the figure and a full table of the whole CNV region, accessions, improvement status, and copy numbers data.

In the detail viewing page, the distribution of the improvement status in different copy numbers is presented in a bar plot fashion for the selected CNV region (Figure 7). From the bar plot, users can visualize the distributions and uncover significant improvement status that links with a particular copy number if possible. At the bottom of the figure, there is a summary table that summarizes the counts of accessions by improvement status and copy numbers. Within the table, the percentages of accession counts are also calculated out of total accessions. Apart from the figure and summary table, a full table with information such as CNV region, CNV region length, accessions, improvement status, and copy numbers is also provided.

**FIGURE 8:**
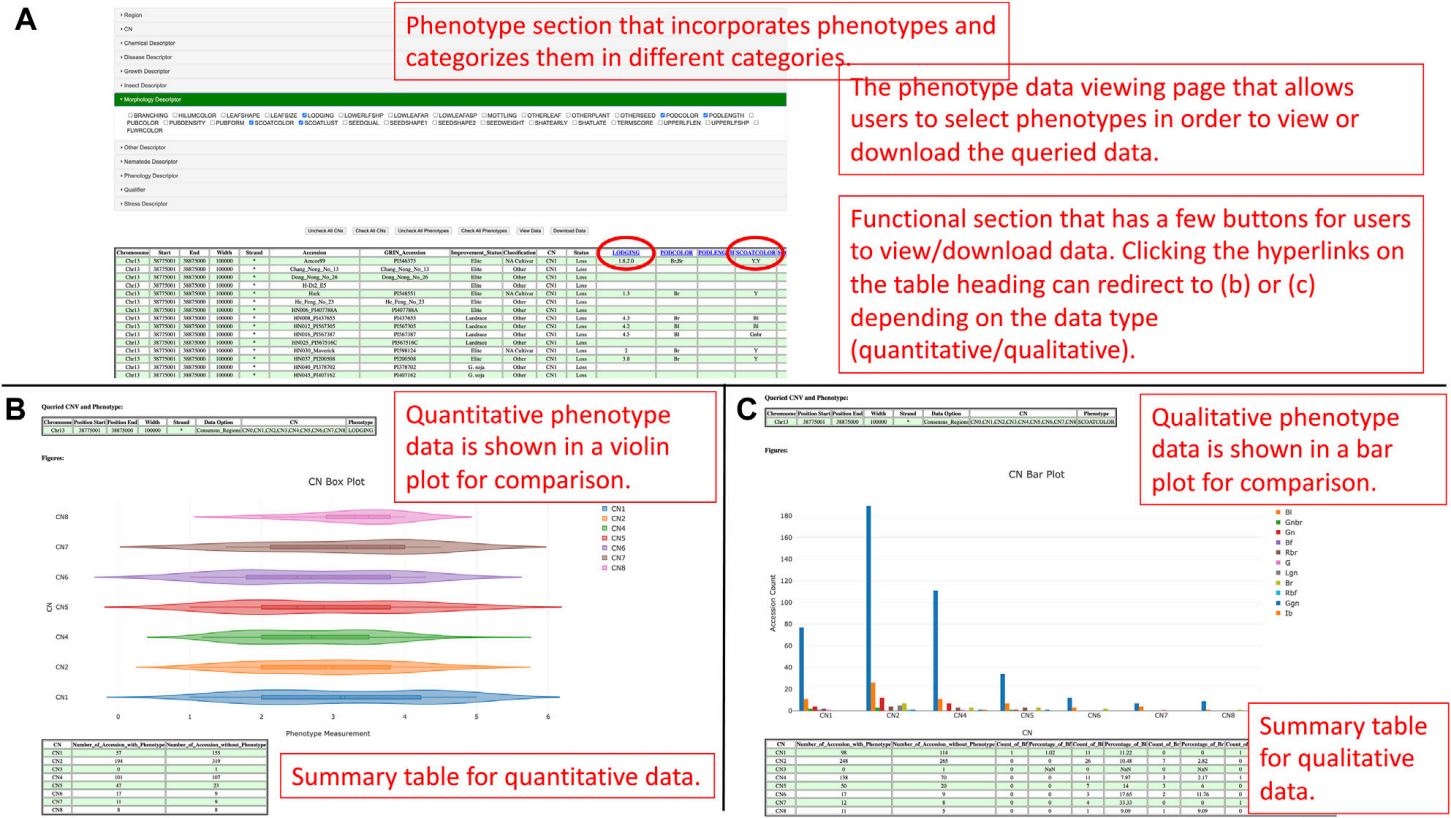
**(A)** The phenotype data viewing page for users to select copy numbers and phenotypes they are interested in and view or download the data. **(B)** The distribution of a quantitative trait is plotted against copy numbers in a violin plot figure. **(C)** The distribution of a qualitative trait is plotted against copy numbers in a bar plot.

If users would like to gain information about accessions and phenotypes, they can click the connect phenotypes button to redirect to the phenotype data viewing page (Figure 8A). On the phenotype data viewing page, users can select the copy numbers of interest and click the view data button for seeing the data or the download data button to collect the data in a comma-separated values (CSV) file format. Additionally, users can also select copy numbers and phenotypes of interest to overlap and view the data. The data in a tabular fashion allows users to click on a phenotype heading for plotting the distributions of the corresponding phenotype data in a violin plot or a bar chart depending on the data type (quantitative or qualitative) (Figures 8B, C).

In a violin plot, users can visualize the quantitative distributions of the phenotype data by different copy numbers. If users hover the pointer on a box in the figure, they can visualize the maximum, minimum, first quantile, third quantile, mean, and median values of that box. Users can also show and hide copy numbers by toggling the elements in the legend. In a bar plot, users can visualize and compare between counts of qualitative categories in the phenotype data by copy numbers. Similarly, users can also toggle elements in the legend to show or hide categories. At the bottom of the violin plot or bar plot, there is a summary table that summarizes the count of accessions by copy numbers. The summarization for quantitative data is counts of accessions by the existence of phenotype data, whereas the summarization for qualitative data is counts and percentages of accessions of each qualitative category. The last figure on the page is a bar plot that shows the distribution of the improvement status of accessions based on selected copy numbers. The figure aims to provide a linkage between the phenotype, improvement status, and copy numbers so that users can understand the improvement status that leads to the phenotype patterns by copy numbers.

### CNV analysis component—Search by Accession and Copy Numbers

The Search By Accession and Copy Numbers section of the CNV analysis component has an accession input box, a copy number input box, a data option dropdown menu, and a search button in a window (Figure 5B). Users can input only one accession into the accession input box, input multiple copy numbers into the copy numbers input box (each in a new line), and select either consensus regions or individual hits in the data option dropdown menu. Upon completing all the fields in the window, users can click on the search button so that queries can be performed and rendered on the results page.

**FIGURE 9:**
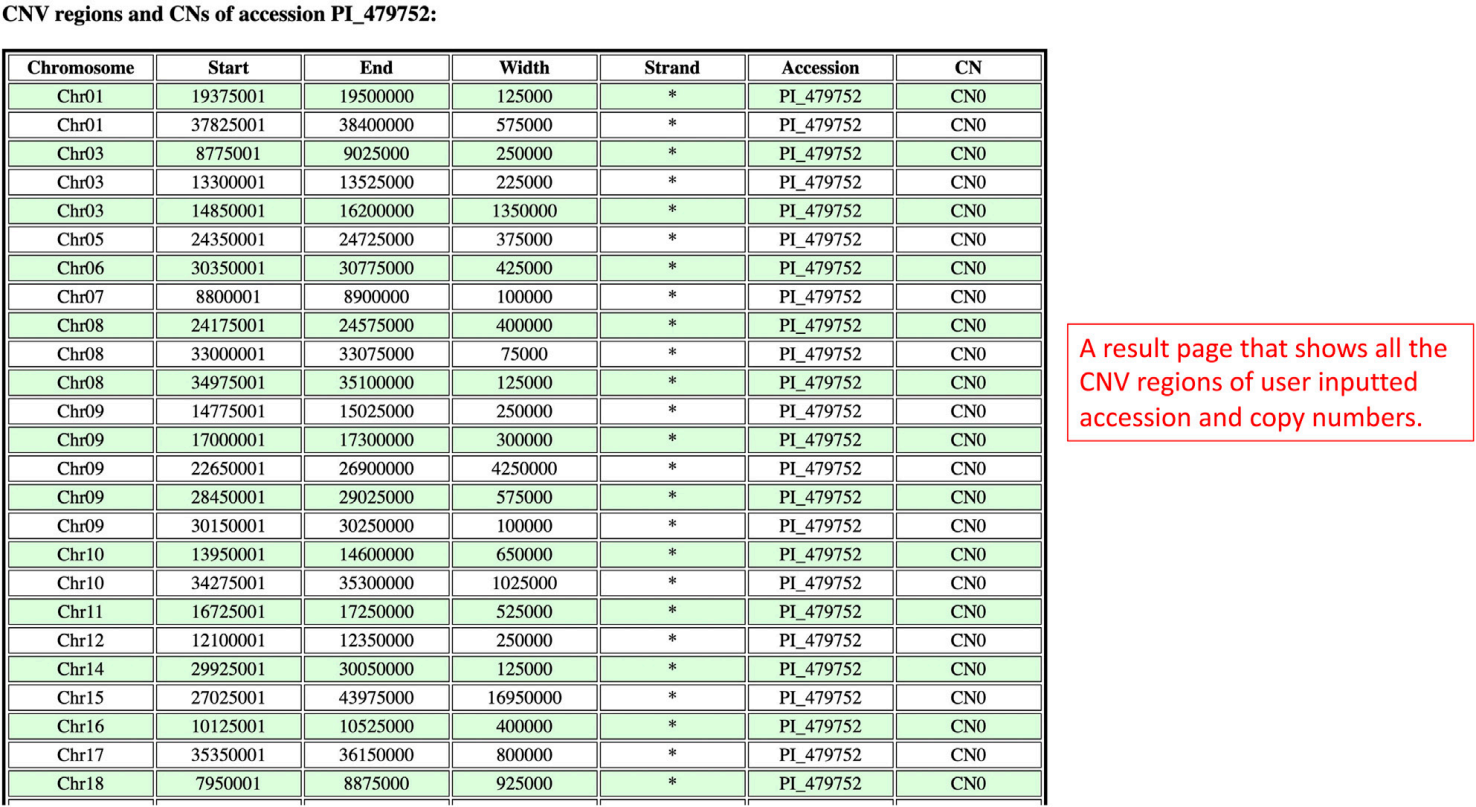
The results page redirected from the Search By Accession and Copy Numbers window to show CNV regions related to the inputted accession and copy numbers.

On the results page, there is only one table that has the information related to the inputted accession and copy numbers (Figure 9). The CNV regions on the table have details such as the region chromosome, region start, region end, width, strand, accession, and copy number. The purpose of this table is to show users all the CNV regions of a particular accession and copy numbers.

### CNV analysis component—Search by Chromosome and Region

The Search by Chromosome and Region section of the CNV analysis component has one window that consists of a chromosome input box, a starting position input box, an ending position input box, a data option dropdown menu, and a search button (Figure 5C). Users can input the region of interest with chromosome, starting position, and ending position into the respective input boxes and select either consensus region or individual hits in the data option dropdown menu. When users are ready to perform queries, they can click the search button, and, simultaneously, the users are redirected to the results page.

**FIGURE 10:**
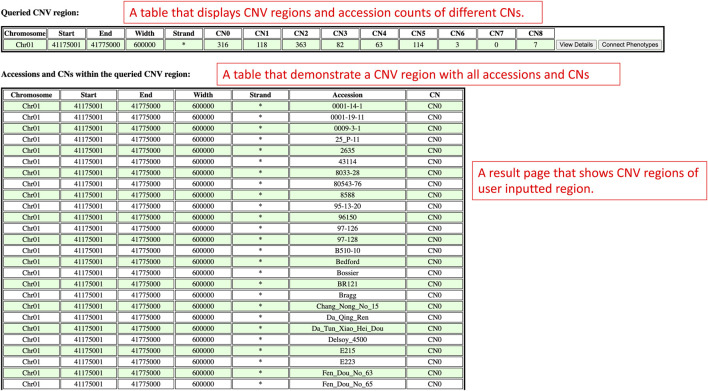
The results page redirected from the Search by Chromosome and Region window to display CNV regions between users’ region of interest.

On the results page, there are two sections which are the queried CNV regions and accession counts section, and the accessions and copy numbers within the queried CNV region section (Figure 10). The queried CNV regions and accession counts section shows a table that contains CNV regions that are bounded between the region of interest input by users. In this section, the table also displays the accession counts in each copy number within each CNV region. At the end of the table, users can also access the view details page and phenotype data viewing page with buttons to understand the improvement status distribution in that CNV region or connect copy numbers and accessions with phenotype data. In order to show more details of each CNV region, the accessions and copy numbers within the queried CNV region section present each CNV region along with all the accessions and copy numbers of that CNV region in a table. If users are interested in knowing the copy number variations within a region of interest, the results presented on this results page will suit their needs.

### Case studies

Analysis of CNV distribution by improvement status offers insight into soybean domestication-related gene gain that impacts plant height.

**FIGURE 11:**
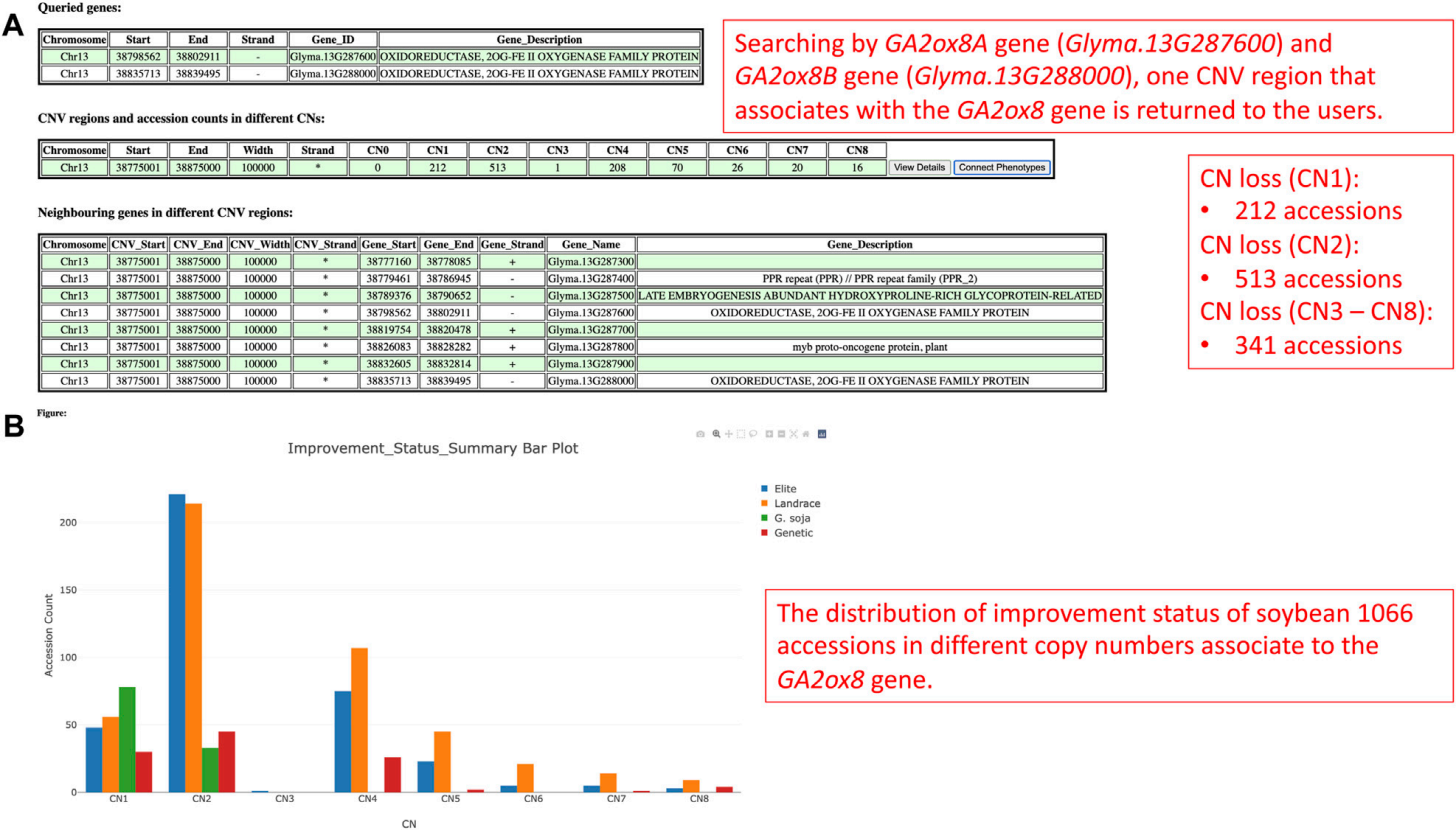
**(A)** The CNV region on chromosome 13 that is, associated with the GA2ox8 gene. **(B)** The soybean 1066 accessions’ improvement status distribution in different copy numbers.

**FIGURE 12:**
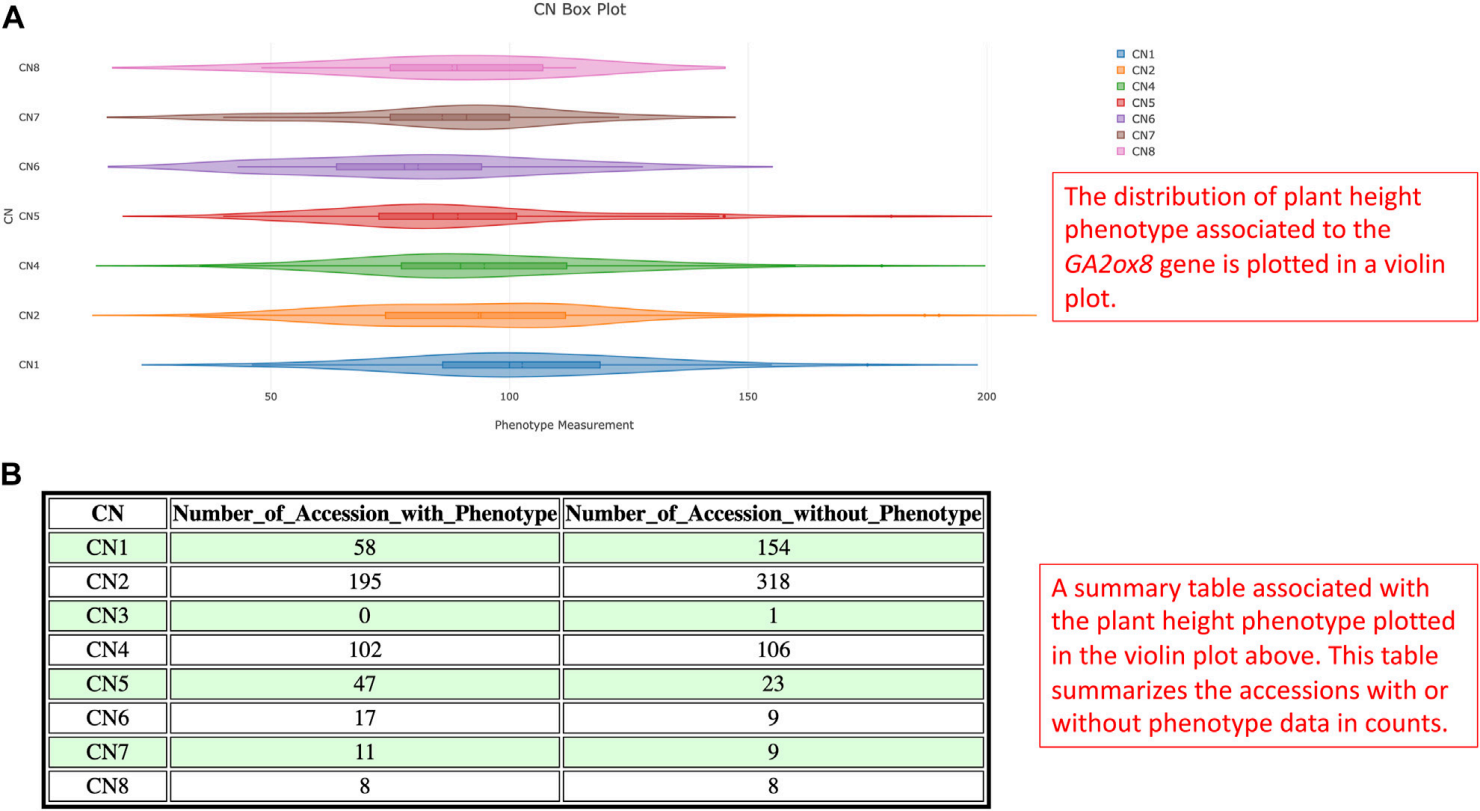
**(A)** The violin plot illustrates the statistical distribution of plant height of soybean 1066 accessions for CNV in the GA2ox8 gene-associated region on chromosome 13. **(B)** A summary table associated with the violin plot documents the corresponding phenotype counts and missing information.

Gibberellin acid oxidase 2 (*GA2ox*) is an enzyme that, besides other enzymes, converts bioactive phytohormone gibberellins (GA) into inactive forms (Thomas et al., 1999). Modulation of GAs metabolism genes played an important role in the green revolution of crop improvement. In soybean, reducing trailing growth and shoot length were observed in soybean *GA2ox8* overexpressing mutants. Furthermore, plant height of wild *G. soja* (*Glycine soja*) ancestor W05 was associated with less copy number of *GA2ox8A* (*Glyma.13G287600*) and *GA2ox8B* (*Glyma.13G288000*) in comparison to generally shorter cultivated *G.* max *C08* (Wang et al., 2021). Thus, this suggests an important role in the *GA2ox8* copy number increase during domestication. Here, we analyzed CNV in the *GA2ox8*-related cn. MOPS predicted CNV associated region, that is, shown in our data on chromosome 13 (Chr13:38,798,562-38,802,911). There are 513 accessions with normal CN, 212 accessions with CN loss, and 341 accessions with CN gain (Figure 11A). Figure 11B illustrates the distribution of CNVs by improvement status in the soybean 1066 accessions and demonstrates that all G. soja accessions possess either CN1 or CN2 that are considered as loss or normal CNV whereas accessions with the other improvement status are all *G.* max (*Glycine max*) and can potentially bear more *GA2ox8* copies. This result is in accordance with Wang et al. (2021) and thus, supports the hypothesis of the *GA2ox8* gain during soybean domestication (Wang et al., 2021). We further associated the observed CNV with soybean plant height phenotype (Figure 12A). Since there is no phenotype data available for any G. soja accessions for plant height in the GRIN database, the conclusions made based on this analysis might be influenced by this fact. However, when comparing the three CNVs with the highest accession counts/known phenotype (CN2 - normal, CN1—loss, and CN4—gain, Figure 12B) here we can see median shifts to increase plant height of accessions with CNV loss (CNV1) in comparison to slightly reduced plant height of normal CNV (CNV2) and CNV gain (CNV4). Most importantly, among 208 accessions with CN4, 141 are Elite accessions with 83 out of the 102-known phenotype accessions in this group. Thus, this result indicates that the *GA2ox8* CN gain might be responsible for plant height in cultivated soybean varieties as demonstrated by Wang et al. (2021); Wang et al. (2021).

## Discussion

The GenVarX toolset offers tools that enable online analyses of the associations of pre-defined phenotypes with their variations in promoter regions and CNV on soybean, rice, and *Arabidopsis*. The toolset provides query, association testing, visualization, and download capabilities for users to obtain new insight from the promoter and CNV data. Using the toolset, users can interact with the web interfaces to do their research without the need of spending long-time processing and running big data to gain promoter and CNV results. It also provides opportunities for users who do not have large-scale servers and large storage space to have access to the promoter and CNV data.

In the development of the GenVarX toolset, we faced some challenges in SNP data processing and phenotype data retrieval. SNP data in a VCF file usually consists of many positions and accessions in a single file. Processing the SNP data in a VCF file and uploading the processed SNP data into a database are usually time-consuming processes. A divide-and-conquer strategy is usually required in code implementation to achieve a certain speed-up in the processing and uploading. Furthermore, multi-processing and multi-threading can also be helpful when processing and uploading the data programmatically. Besides that, retrieving the SNP data from the database could also be a slow process. Therefore, a database indexing method is required to increase the data retrieval speed. Apart from the SNP data processing problem, we faced another challenge that was caused by the limited availability of phenotype data for rice and *Arabidopsis*. To solve this problem, online data explorations are required in order to find suitable data for our GenVarX toolset.

The GenVarX toolset is developed with the incorporation of extensive capabilities that are related to genotype and phenotype. The extensions of showing gene binding sequences, sequence logo figures, mutative variant positions, as well as the linkage of the genotype data to phenotype data are the strengths of this toolset. Although PlantTFDB and PlantRegMap are the main sources for the datasets of the GenVarX toolset, these new capabilities are not developed in their web portals. Another promoter database is the Eukaryotic Promoter Database (EPD) (Périer et al., 2000) which also allows users to search for promoters. However, important crop species like soybean and rice are not available in that database. In terms of CNV, there is a lack of plant and crop related CNV databases for users to perform queries, visualize CNV, and link CNV to phenotypes. Thus, our research group developed the GenVarX toolset to assist the research community to advance their research.

In future development, our research group will focus on expanding the GenVarX toolset to support more organisms. Besides that, we will also incorporate more phenotypic data into the database to allow users to visualize different phenotypes more easily. Furthermore, integration of CNV results from other tools can also be done to make the CNV component of the GenVarX toolset capable of a more enriched comparative analysis. As mentioned in Gabrielaite et al., which shows the comparisons of different CNV tools in terms of outcomes, metrics, and performance, there are several tools such as GATK gCNV, Lumpy, and DELLY, that were developed with different methodologies that outperform many other CNV tools (Gabrielaite et al., 2021). These CNV tools can be used to further analyze our data and integrate into the GenVarX toolset so that users can select CNVs from different methods to link with genotype and phenotype.

## Conclusion

In the GenVarX toolset development, we have collected and processed publicly available data from various sources and platforms. Having the data, we have built the GenVarX toolset that has promoter regions and CNV analysis components for soybean, rice, and *Arabidopsis*. The soybean GenVarX toolset is deployed on the SoyKB website (https://soykb.org/SoybeanGenVarX/) whereas the universal GenVarX toolset for other organisms is deployed on the KBCommons website (https://kbcommons.org/system/tools/GenVarX/Osativa and https://kbcommons.org/system/tools/GenVarX/Athaliana). The broad plant research community can utilize the GenVarX toolset as a comprehensive source of information to gain insights into soybean, rice, and *Arabidopsis* promoter regions or CNV analysis outcomes. More specifically, a better understanding of variations in the TF binding sites and CNV can be predicted with the toolset. Hence, it serves as a valuable pre-experimental step for further gene transcription studies.

## Available and requirements

Project Name: GenVarX

Project Homepage:

• Soybean GenVarX Toolset: https://soykb.org/SoybeanGenVarX/
• Rice GenVarX Toolset: https://kbcommons.org/system/tools/GenVarX/Osativa
• *Arabidopsis* GenVarX Toolset: https://kbcommons.org/system/tools/GenVarX/Athaliana

Programming Languages:

• Data Analytics: Python and R
• Web Development: PHP, HTML, CSS, and JavaScript

Other Requirements:

• Data Analytics:
  o Python 3.7.0 or higher
  o R 3.6.0 or higher
  o SQLAlchemy 1.4.41 or higher
  o cn.MOPS 1.40.0 or higher
  o Burrows-Wheeler Aligner (BWA) 0.7.17
  o Genome Analysis Toolkit (GATK) 4.2.6.1
• Web Development:
  o PHP 8
• Web Browsing:
  o Google Chrome (Recommended), Firefox, or Microsoft Edge

Source Code:

• GenVarX Data Processing Scripts: https://github.com/yenon118/GenVarX_Data_Processing
• Soybean GenVarX Toolset Source Code: https://github.com/yenon118/SoybeanGenVarX
• Rice and *Arabidopsis* Toolsets Source Code: https://github.com/yenon118/GenVarX

License:

• Soybean GenVarX Toolset: MIT License
• Rice GenVarX Toolset: MIT License
• *Arabidopsis* GenVarX Toolset: MIT License

## Data Availability

The original contributions presented in the study are included in the article/Supplementary Material, further inquiries can be directed to the corresponding authors.

## Abbreviations

BAM, binary alignment map; BWA, burrows-wheeler aligner; ENA, european nucleotide archive; GATK, genome analysis toolkit; GSA, genome sequence archive; GRIN, resources information network; Indels, insertions and deletions; NCBI, national center for biotechnology information; SAM, sequence alignment map; SNP, single nucleotide polymorphism; VCF, variant call format; WGRS, whole genome re-sequencing.
